# Integrated approaches to target nuclear receptors for managing the co-morbidity of tuberculosis and diabetes

**DOI:** 10.3389/fimmu.2026.1824354

**Published:** 2026-06-10

**Authors:** Rashmi Arora, Nancy Ahuja, Saumyata Kumawat, Vipashu Kaushal, Rahul Sharma, Pawan Gupta

**Affiliations:** 1 Department of Molecular Biology, Council of Scientific and Industrial Research, Institute of Microbial Technology, Chandigarh, India; 2 Academy of Scientific and Innovative Research (AcSIR), Ghaziabad, Uttar Pradesh, India

**Keywords:** combination therapy, co-morbidity, nuclear receptors, tuberculosis, type 2 diabetes

## Abstract

**Introduction:**

The dual epidemic of tuberculosis (TB) and type 2 diabetes mellitus (T2DM) presents a critical global health challenge, as diabetic immunosuppression increases TB susceptibility while TB infection exacerbates glucose intolerance. Because nuclear receptors (NRs) regulate both metabolic pathways and infectious disease responses, they represent promising therapeutic targets. This study aimed to evaluate the therapeutic potential of targeting overlapping NRs to manage TB-T2DM comorbidity.

**Methods:**

A comorbid mouse model was established by inducing T2DM via a high-fat diet and streptozotocin, followed by an aerosol challenge with Mycobacterium tuberculosis (M. tb). Mice were categorized into three groups: uninfected controls, M. tb-infected non-diabetic, and M. tb-infected diabetic mice. NR expression profiling was performed on alveolar macrophages, specifically screening endocrine, adopted orphan, and orphan NRs. Based on this profile, comorbid mice were treated with a combination therapy (CT) consisting of specific ligands for the most promising NR targets Vdr, Lxr and Rev-erbα.

**Results:**

Expression screening identified Vdr, Rev-erbα, and Lxr as key dysregulated receptors with dual roles in TB and T2DM pathogenesis. Administration of the triple-ligand CT to comorbid mice significantly alleviated both metabolic and infectious symptoms compared to untreated comorbid controls. Specifically, CT-treated mice demonstrated reduced T2DM severity (stabilized body weight, decreased blood glucose, and lowered glycated hemoglobin) alongside reduced TB disease burden, evidenced by lower bacterial colony-forming unit (CFU) counts and fewer pulmonary granulomatous lesions.

**Discussion:**

These findings demonstrate that simultaneous modulation of Vdr, Rev-erbα, and Lxr effectively mitigates the severe manifestations of TB-T2DM comorbidity. Integrating metabolic and antimicrobial treatments via host-directed nuclear receptor therapies offers a potent, novel strategy to combat this complex dual epidemic, particularly in high-prevalence regions.

## Graphical Abstract

**Figure:**
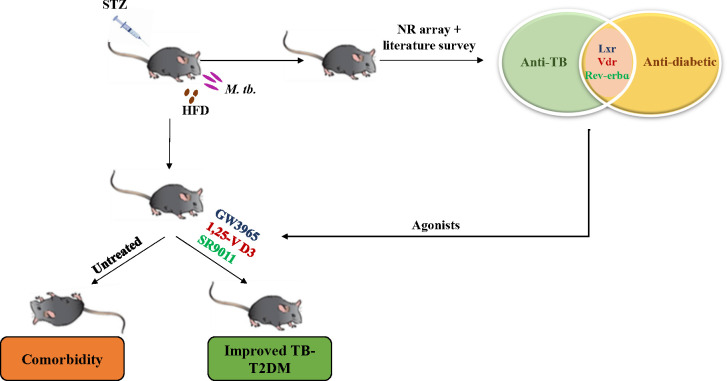
Tuberculosis (TB) and diabetes are induced in mice using *M. tb* and a combination of HFD+STZ, respectively. Through a NR array and literature review, it was identified that Lxr, Vdr, and Rev-erbα exhibit both anti-TB and anti-diabetes effects. Consequently, mice were treated with the ligands for these receptors in combination, resulting in improved TB- T2DM condition.

## Introduction

Diabetes Mellitus (DM) is a chronic metabolic condition in which the pancreas produces insufficient insulin or the body does not utilize the insulin effectively. The International Diabetes Federation (IDF) estimates that in 2021, 536.6 million adults in the 20–79 years are living with diabetes (1). Since this number is predicted to increase to an additional 783.2 million by 2045 with a prevalence of 12.2%, DM is proving to be a burden on worldwide public health (2). It can be classified into three types: type 1 diabetes mellitus (T1DM), an autoimmune disorder that destroys the pancreatic β-cells; type 2 diabetes mellitus (T2DM), characterized by insulin resistance; and gestational diabetes.

Numerous research studies have discovered that DM elevates the susceptibility to TB, and conversely, individuals with TB exhibit elevated rates of developing DM (3–5). Tuberculosis (TB) can cause diabetes by triggering systemic inflammation and immune responses that interfere with insulin signaling, primarily through proinflammatory cytokines like IL-1β, IL-6 and IL-18, alongside anti-inflammatory cytokines like IL-10 (6). TB-induced changes in adipose tissue, such as increased lipolysis and altered adipokine secretion, lead to elevated free fatty acids that contribute to insulin resistance (7). Additionally, TB disrupts glucose and lipid metabolism and reduces amino acid levels, further impairing the body’s ability to regulate blood sugar (7). The risk of contracting TB is tripled due to DM, and DM is also associated with unfavorable outcomes in TB treatment, including increased mortality rates. In individuals with DM, compromised immune responses contribute to either the onset of active TB infection or the reawakening of latent TB. Immunity is weakened by persistent hyperglycemia (both innate and adaptive). Poor glycemic control impacts cytokine response and changes alveolar macrophage (AMs) defenses, and DM impedes cell-mediated immunity (8).

Consequently, tailored care and treatment approaches are imperative for effectively managing both conditions, especially in regions where both DM and TB prevalence are high. Various studies have demonstrated the role of nuclear receptors (NRs) as the druggable target in metabolic and infectious diseases. NRs are reported in literature to mitigate DM as well (9, 10).

TB has been increasingly linked to NRs that modulate host immune and metabolic responses (11–17). In our previous studies, as well as those of others, three NRs, reverse of the erb alpha (Rev-erbα) (NR1D1), liver X receptor (Lxr), and vitamin D receptor (Vdr) have been identified for their potential to alleviate TB (18–20). Numerous reports highlight the critical roles of these receptors to play a role in DM (9, 21, 22).

To model the synergistic confluence of TB and T2DM, we employed a non-genetic high fat diet (HFD) + low-dose streptozotocin (STZ) protocol. This model is advantageous over genetic models as it mimics the polygenic and environmental nature of human T2DM. By combining HFD-induced insulin resistance with a low-dose STZ (40 mg/kg) injection to induce moderate β-cell impairment, we avoided the rapid apoptosis associated with Type 1 models, instead achieving a chronic state of T2DM-like metabolic dysfunction (23). This approach is highly reproducible and consistent with previous studies in similar experimental settings (24).

We generated an expression atlas of NRs from AMs in TB– T2DM mice and observed significant changes in the expression levels of Rev-erbα, Lxr, and Vdr. While current treatments independently address either TB or DM, there is a significant lack of integrated host-directed therapies capable of managing the synergistic complications of both diseases. Consequently, this study aimed to evaluate the therapeutic potential of a combinatorial approach targeting three specific NRs. We hypothesized that the simultaneous modulation of these receptors would provide a multifaceted defense by enhancing antimicrobial immunity while concurrently stabilizing metabolic dysregulation in the TB- T2DM comorbid state.

In this study, we have developed a comorbid model of TB- T2DM after feeding the mice with HFD and STZ injection followed by aerosol infection with H37Rv (25). After the infection has been established, mice were treated with SR9011, GW3965 and 1, 25 dihydroxyvitamin D3 which are the ligands of Rev-erbα, Lxr and Vdr respectively in the form of CT to unveil its effect on TB- T2DM coexisting ailment (26–28).

Body weight, blood glucose and glycated hemoglobin were used as index to check T2DM whereas colony forming units (CFU) count and histopathology were the parameters to determine the bacterial load. Targeting these NRs for the prevention and management of both TB and T2DM co-morbidity in prevalent regions is discussed.

## Material and methods

### Generation of diet induced murine model of T2DM

Experiments with mice were approved by the Institutional Animal Ethics Committee of the Institute of Microbial Technology and performed according to the National Regulatory Guidelines issued by the Committee for the Purpose of Supervision of Experiments on Animals (Number 55/1999/Committee for the Purpose of Control and Supervision of Experiments on Animals (CPCSEA)), Ministry of Environment and Forest, Government of India. At 4 weeks of age, mice were divided into two groups. One group of mice received standard rodent diet and other group was fed with high-fat diet along with three intraperitoneal injections of 40 mg/kg streptozotocin on consecutive days to generate non genetic mouse model of T2DM. Mice were fasted for 12 hours before having their blood glucose levels checked using an Accu-Chek blood glucose meter. Mice were classified as diabetic if fasting blood glucose is >200 mg/dl (23).

### Aerosol infection

Experimental mice were exposed to aerosol inhalation of *M. tb* H37Rv (∼100 CFU per lung) using the inhalation exposure system (Glas-Col; Terre Haute, IN) in the BSL3 facility, at IMTECH as described previously. Mice were allowed to establish the infection for 30 days and later sacrificed and AMs were isolated (29).

### PCR array

RNA was isolated from cells using a RNeasy Mini Kit (Qiagen). cDNA was synthesized from 1 µg of total RNA using an RT^2^ First Strand Kit (Qiagen) followed by RT-qPCR using RT^2^ SYBR Green ROX qPCR Mastermix (Qiagen). Customized PCR Array plates (Qiagen) were used to ascertain expression profile of all the murine NRs and associated co-regulators as per manufacturer’s instructions (30). Relative fold regulation was determined by using 2^-ΔΔCt^ method. The NRs with a Ct value less than 35 were only included in the analysis.

### Histopathology

Lung tissues were fixed in a 10% buffered formalin solution. After fixation, the tissue was embedded in paraffin and microtome sections were stained with hematoxylin and eosin (H&E) stain. The pictures were captured at a magnification of 100X and scale bar was added using ImageJ according to standard protocol. When samples were sent for histopathological investigation, the identities of the samples were masked.

### *M. tb* survival assay

Mice were exposed to aerosols of *M. tb* H37Rv to establish an initial infection of 100 CFU at 24 hours. Bacterial load was enumerated. Two weeks post-infection, a distinct set of mice was sacrificed to quantify bacterial multiplication and establish the baseline bacterial load prior to therapy. At the same time point (2 weeks post-infection), the remaining mice were randomized into experimental cohorts to receive either the CT or the vehicle control. The animals received intraperitoneal injections of 10 ng/kg of 1, 25 dihydroxyvitamin D3 for Vdr, 33 mg/kg of SR9011 for Rev-erbα and 6.6 mg/kg of GW3965 for Lxr twice per week. The animals were assessed for bacterial load, 2, 4, 6 and 8 weeks post-treatment.

### Enzyme-linked immunosorbent assay of glycated hemoglobin

Glycated hemoglobin levels were quantified in lysed RBCs isolated from blood of TB- T2DM and TB- T2DM along with combined treatment, using commercial ELISA kits specific for mice hemoglobin according to the manufacturer’s instructions.

### Statistical analysis

Graph pad prism was used for statistical analysis. The results are expressed as mean ± SD unless otherwise mentioned. For comparisons between two experimental groups, a two-tailed Student’s t-test was employed. Statistical significance is indicated in the figures using specific symbols to distinguish between comparison types: asterisks (*) denote comparisons with the control group, while hashtags (**#**) denote comparisons within treated groups or between specific cohorts as indicated. The following thresholds for statistical significance were set: *, #*P* < 0.05; **, ##*P* < 0.01; and ***, ###*P* < 0.001. The number of animals for each experiment (n) is indicated in the respective figure legends.

## Results

### Induction of diet induced murine model of T2DM

**Figure 1:**
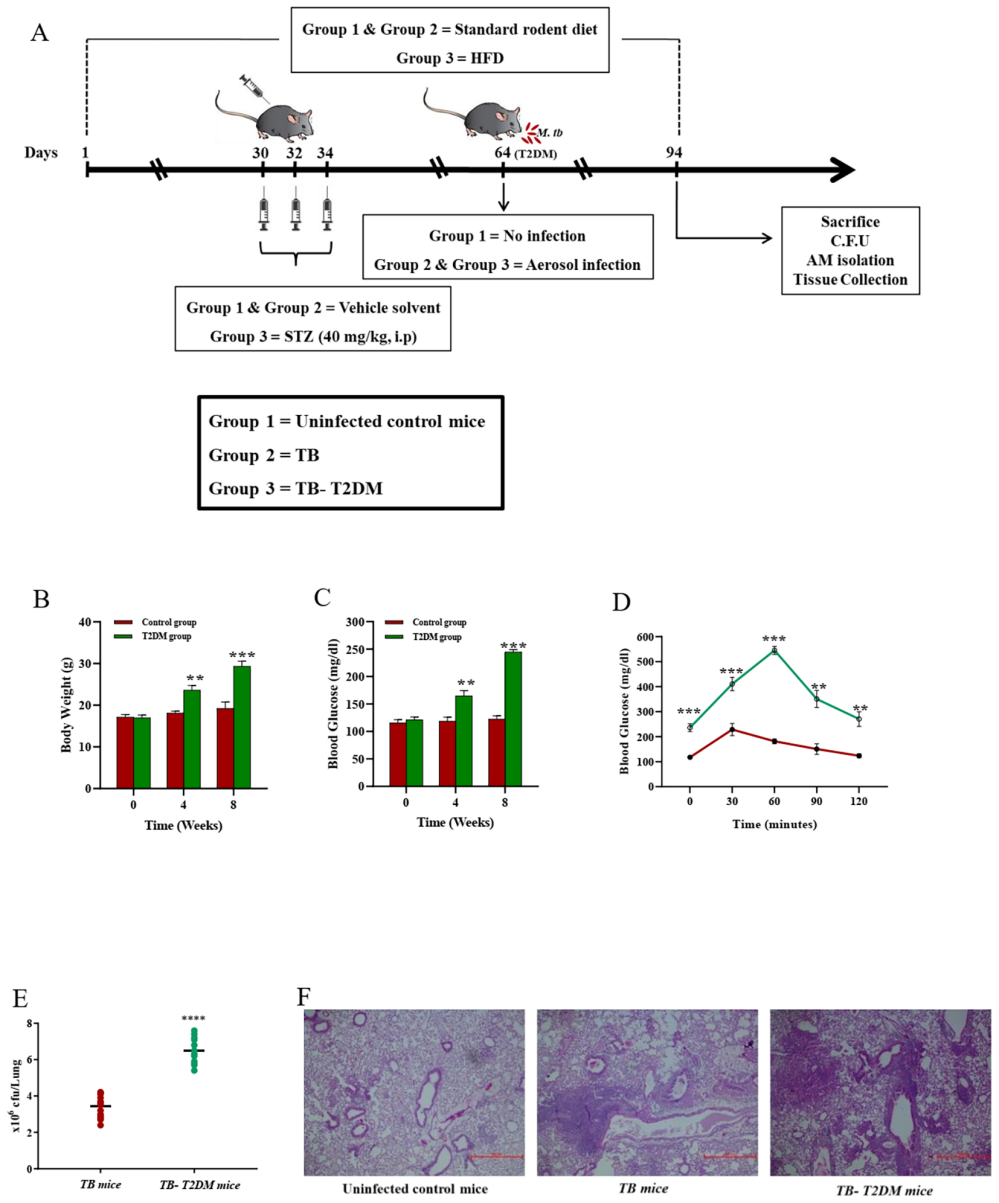
Development of HFD induced T2DM mice model. **(A)** Schematic of experimental schedule **(B)** Body weight changes in standard rodent diet (n=6) and HFD fed mice (n=6) were monitored in the beginning, at week 4 and at week 8 of HFD feeding. **(C)** Fasting blood glucose levels were monitored at similar time points. **(D)** Glucose tolerance test was performed at week 8 of HFD feeding. **(E)** On day 30 of infection, CFU count was measured after an aerosol challenge with *M. tb* to investigate *M. tb* survival in non-diabetic (n=6) and diabetic mice (n=6). Horizontal lines represent the mean. **(F)** Histopathology of uninfected control mice, TB and TB- T2DM mice lungs at day 30 after infection. Data are mean ± SD from three independent experiments (n = 6 mice per group per experiment). ** P < 0.01, *** P < 0.001 compared to control group by student t-test.

To generate non genetic mouse model of T2DM, male C57BL/6 mice, at 4 weeks of age were divided into two groups. One group (non-diabetic mice) received standard rodent diet and other group (diabetic mice) was fed with HFD. Composition of HFD is given in **Supplementary Table 1**. After four weeks of HFD, mice were given three intraperitoneal injections of 40 mg/kg STZ for three alternate days. They received the HFD for additional four weeks. The schematic of the mice model generation is illustrated in **Figure 1A**. Body weight and blood glucose levels were measured. There was increase in body weight of HFD fed mice as compared to non-diabetic mice who received standard diet (**Figure 1B**). Fasting blood glucose levels were more than 200 mg/dl in HFD fed mice (**Figure 1C**). To assess glucose homeostasis, glucose tolerance test (GTT) was performed. Diabetic mice showed significantly impaired glucose clearance as compared to non-diabetic mice (**Figure 1D**). Hence, T2DM mice model was successfully generated with diabetic mice group showing metabolic characteristics of T2DM.

To generate combined model of TB and T2DM, non-diabetic mice and diabetic mice were aerosol challenged with H37Rv. After 30 days of infection, the lung bacterial burden was determined by CFU assay. Mice with comorbid conditions exhibited a significantly higher CFU count compared to non-diabetic infected mice (**Figure 1E**). Histopathological parameters were determined by H&E staining and lung sections were examined from the respective animals. There were no signs of inflammation in lung sections of uninfected control mice whereas well-formed granulomas were observed in TB and TB- T2DM mice. TB- T2DM mice developed severe peribronchiolitis and more granulomatous lesions as compared to TB mice (**Figure 1F**).

### Expression profiling of NRs in AMs isolated from TB and TB- T2DM mice

**Figure 2:**
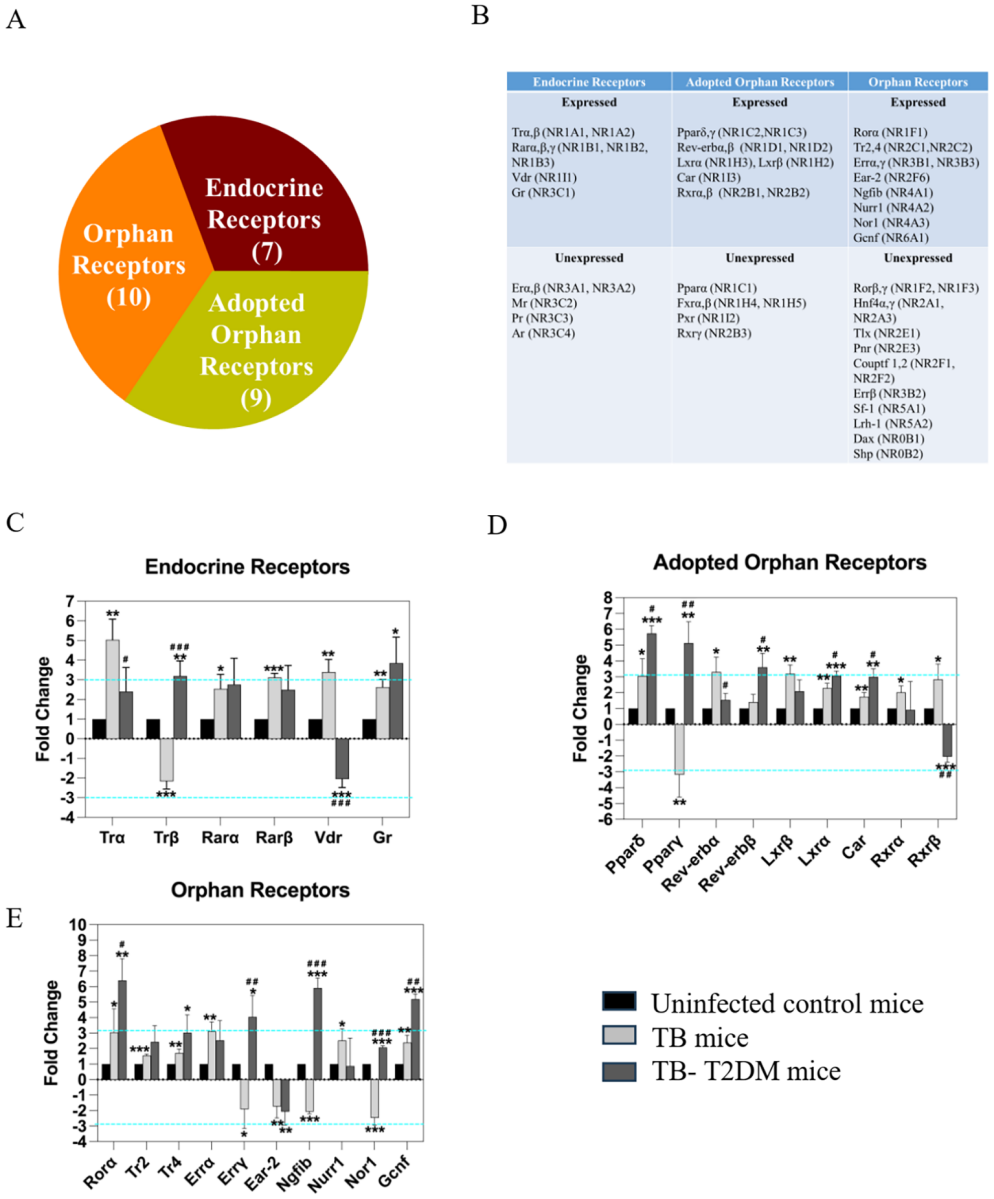
Composition of NRs expressed in AMs. **(A)** 26 out of 49 murine NRs found expression in AMs including 7 endocrine receptors, 9 adopted orphan receptors and 10 orphan receptors. **(B)** NRs that are expressed or not expressed in AMs are given in a table with their unified nomenclature system names in parenthesis. If the cycle threshold (Ct) value of a receptor was more than 35, it was considered unexpressed. Relative expression of **(C)** Endocrine **(D)** Adopted orphan **(E)** Orphan NRs, in uninfected control mice, *M. tb* infected non-diabetic mice and *M. tb* infected diabetic mice. NRs that are up- or down- regulated by at least two-fold in either case are depicted. Data are presented as mean ± SD from three independent experiments (n = 3 mice per experiment). * P < 0.05, ** P < 0.01, *** P < 0.001 shows comparison between uninfected control group vs TB and uninfected control vs TB- T2DM mice; # P < 0.05, ## P < 0.01, ### P < 0.001 shows comparison between TB mice vs TB- T2DM mice.

We challenged T2DM mice and non-diabetic mice by aerosol infection with virulent *M. tb* and isolated AMs at day 30 post infection. We next profiled the transcript levels of NRs in AMs isolated from uninfected control mice, *M. tb* infected non-diabetic mice and *M. tb* infected T2DM mice. The comprehensive examination of the murine NR superfamily revealed that 26 out of 49 known receptors are expressed within AMs. This subset comprises 7 endocrine receptors, 9 adopted orphan receptors, and 10 orphan receptors, all of which exhibited distinct differential expression profiles in our analysis (**Figure 2A**). Table depicting all the NRs which are expressed and unexpressed in AMs are shown in **Figure 2B**. The expression analysis revealed that there was differential expression of some of the NRs in *M. tb* infected non-diabetic mice and *M. tb* infected T2DM mice as compared to uninfected mice. While all receptors showing a fold change greater than 2 are visualized in **Figure 2B**. We focused our subsequent analysis on the subset of NRs exhibiting a highly significant change, defined as a greater than three-fold increase or decrease compared to the baseline. Marked increase in the expression of Trα, Rarβ, Vdr, Pparδ, Rev-erbα, Lxrβ, Rorα, Tr4 and Errα was found in *M. tb* infected non-diabetic mice as compared to uninfected control mice (**Figures 2C–E**).

We then compared the differential expression of NRs in *M. tb* infected T2DM mice and uninfected controls and found that there was increased expression of Trβ, Gr, Pparδ, Pparγ, Rev-erbβ, Lxrα, Car, Rorα, Tr4, Errγ, Ngfib and Gcnf NRs. We next sought to compare the expression changes in *M. tb* infected non-diabetic mice and *M. tb* infected T2DM mice. We discovered that there was notable difference in the expression of Trβ, Vdr, Pparδ, Pparγ, Rev-erbβ, Lxrα, Car, Rxrβ, Rorα, Errγ, Ngfib, Nor1, and Gcnf (**Figures 2C–E**).

Increased expression of Pparδ, Rev-erbβ, Rorα, and Gcnf in *M. tb* infected non-diabetic mice was further upregulated significantly in *M. tb* infected T2DM mice. However, the increased expression of Trα, Vdr and Rev-erbα was downregulated in *M. tb* infected T2DM mice. There were some NRs such as Trβ, Pparγ, Errγ, and Ngfib whose expression was decreased in *M. tb* infected non-diabetic mice while *M. tb* infection in T2DM mice led to their upregulation (**Figures 2C–E**). Additionally, we also postulated that NR does not always need to undergo an expression change to play a functional role in AMs. In this context, TR4 is known to enhance the survival of *M. tb* inside macrophages and hence contributing to TB pathogenesis (29); however, its expression did not remarkably change in *M. tb* infected non-diabetic mice as compared to uninfected mice. Together, these findings showed the NR super-family expression pattern in lung resident AMs isolated from three different groups of mice. Our group, along with others, has demonstrated the individual roles of Rev-erbα, Lxr, and Vdr in modulating TB and T2DM alone. However, the significant changes observed in the fold difference of these NRs in the TB- T2DM comorbidity model prompted us to further investigate their potential involvement in the pathophysiology of TB- T2DM comorbidity.

### Protective effect of CT on T2DM

**Figure 3:**
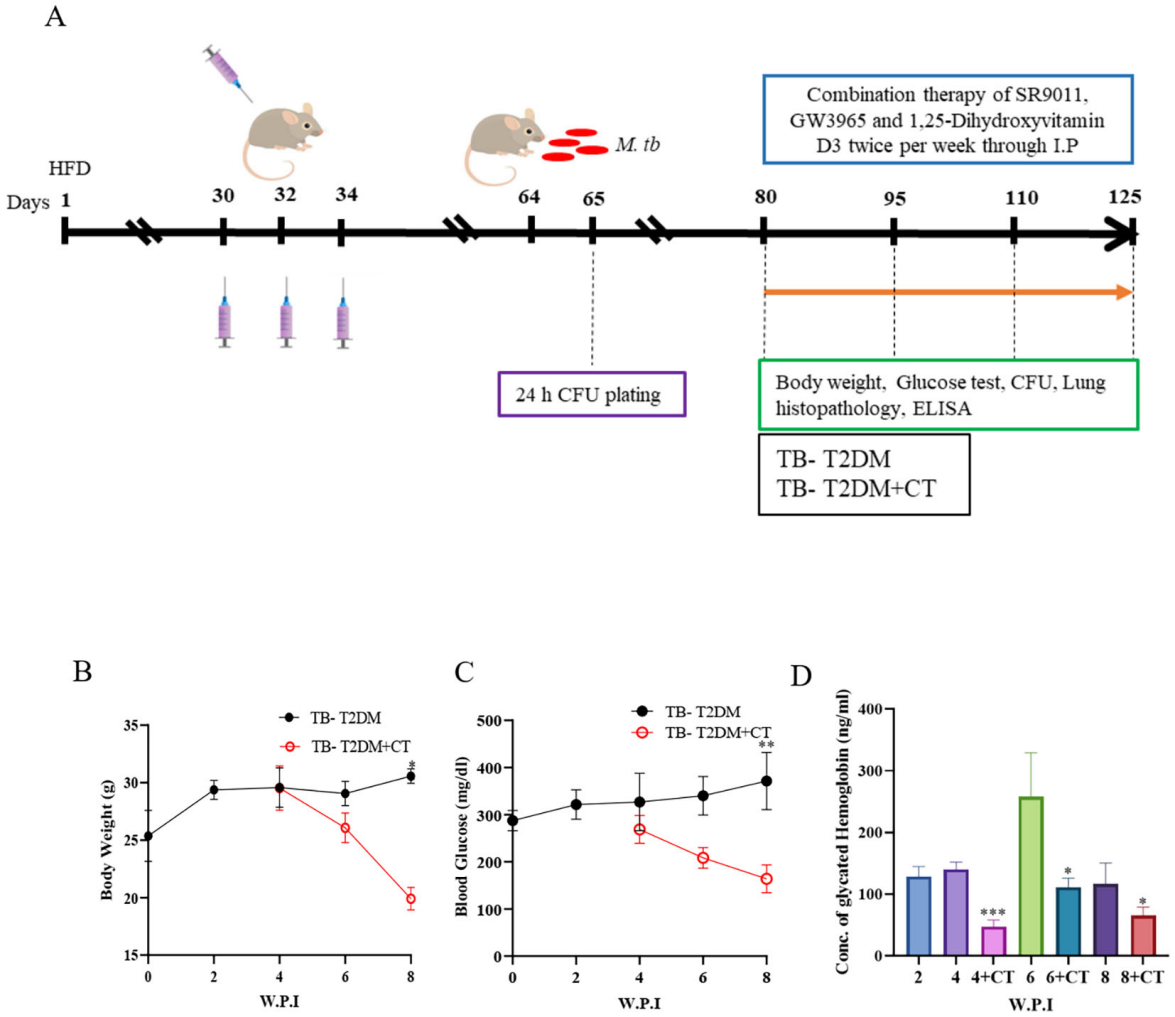
Effect of CT on body weight and glucose levels in TB- T2DM mice model. **(A)** Schematic representation of experimental schedule and drug regimen **(B)** Body weight changes in TB- T2DM mice (n=4) and TB- T2DM mice along with the CT (n=4) were compared in the beginning and at 2, 4, 6 and 8 weeks post infection. **(C)** Fasting blood glucose levels were monitored at similar time points. **(D)** ELISA was performed from lysed RBCs having glycated hemoglobin at 2, 4, 6, and 8 weeks post infection Data are mean ± SD from n = 4 mice per experiment. *P < 0.05, **P < 0.01, ***P < 0.001 compared to TB- T2DM without CT.

TB- T2DM mice (n= 4 per group) were treated with the CT and the other group serves as untreated TB- T2DM. The schematic of drug regimen is illustrated in **Figure 3A**. To evaluate the potential of multi-receptor targeting, we prioritized Rev-erbα, Lxr, and Vdr for combinatorial ligand therapy, as these receptors demonstrated the most significant transcriptional changes in the comorbid model. TB- T2DM mice treated with the combination therapy showed a decrease in the body weight (**Figure 3B**), glucose levels (**Figure 3C**) and glycated hemoglobin (**Figure 3D**) as compared to untreated TB- T2DM mice at 4, 6 and 8 weeks post infection.

### Protective effect of CT on *M. tb* survival

**Figure 4:**
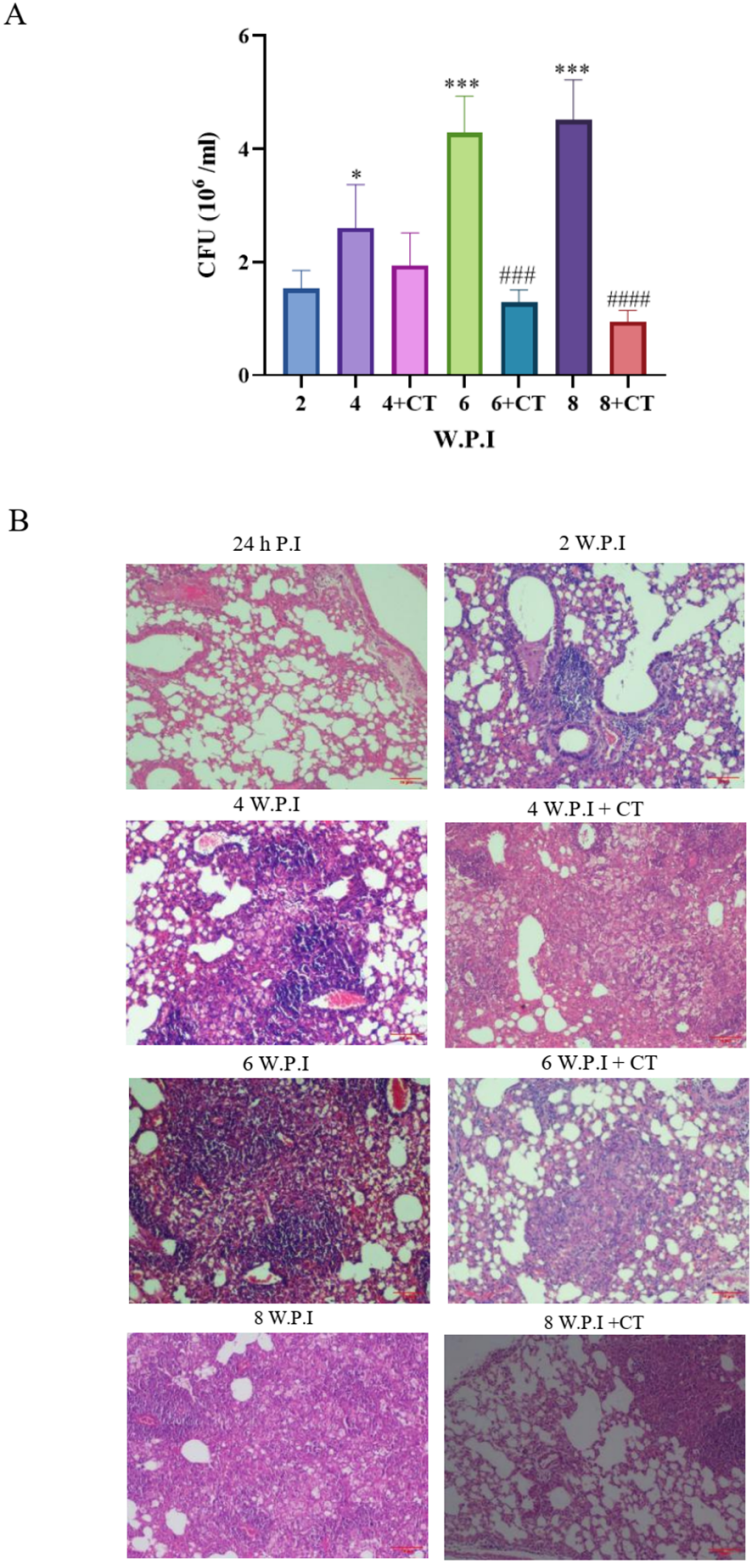
Effect of CT on *M. tb* survival in TB- T2DM mice model. **(A)** CFU count for n=4 per group was measured in TB- T2DM and TB- T2DM with CT at 2, 4, 6, and 8 weeks post infection **(B)** Granulomas were evaluated by histopathology of *M. tb* infected lungs of TB- T2DM mice and TB- T2DM mice along with the CT at 2, 4, 6, and 8 weeks post infection. Data are mean ± SD from n = 4 mice per experiment. *P < 0.05, **P < 0.01, ***P < 0.001 compared to TB- T2DM without CT.

TB- T2DM mice were sacrificed at 2 weeks post infection to quantify the established bacterial load in the lungs prior to any pharmacological intervention. Untreated and mice treated with CT were sacrificed at 4, 6 and 8 weeks post infection (n=4 per group) to check the effect of these ligands on *M. tb* survival. TB- T2DM mice showed decrease in the bacterial load when treated with the CT as compared to the untreated TB- T2DM mice at 4, 6 and 8 weeks after infection (**Figure 4A**). Lungs were isolated and histologically evaluated for granuloma formation. There was a remarkable difference in the histological sections showing less granulomas in CT as compared to lungs of untreated TB- T2DM mice (**Figure 4B**).

## Discussion

The dual burden of TB and T2DM creates a synergistic “syndemic” where metabolic dysfunction impairs immune surveillance, and chronic infection exacerbates insulin resistance. In this study, we demonstrated that a triple-ligand CT targeting Vdr, Lxr, and Rev-erbα significantly reduced bacterial burden and improved metabolic indices (blood glucose and HbA1c) in a murine comorbid model. Unlike standard treatments that address these pathologies in isolation, our findings suggest that simultaneous modulation of these three NRs provides a multifaceted host-directed defense.

Our observation of improved glucose homeostasis and reduced bacterial counts in CT-treated mice is consistent with the known protective roles of the Vdr. Vdr signaling is crucial for preserving β-cell mass and reducing islet inflammation (31). In the context of infection, Vitamin D has been shown to reduce lipid accumulation in foamy macrophages, thereby limiting the intracellular nutrient supply for *M. tb* (32). By including a Vdr ligand in our CT, we likely enhanced the innate antimicrobial response of alveolar macrophages while simultaneously mitigating the STZ-induced inflammatory stress on the pancreas.

The inclusion of an Lxr agonist in our regimen addresses the specific metabolic fuel that *M. tb* exploits. Lxr is a master regulator of cholesterol and lipid metabolism; its agonists have been reported to improve insulin sensitivity and suppress hepatic gluconeogenic genes (9). Our results showing reduced pulmonary granulomatous lesions align with previous studies where Lxr-deficient mice exhibited extreme susceptibility to TB (20, 33). We postulate that Lxr activation in our comorbid model works in tandem with Vdr to promote a leaner macrophage phenotype that is less permissive to mycobacterial persistence.

Rev-erbα serves as a critical link between the circadian clock and immune-metabolic health. Mechanistically, Rev-erbα enhances antimicrobial properties by modulating autophagy and directly repressing the anti-inflammatory cytokine IL-10 (18). Its anti-diabetic role is equally vital, as it suppresses hepatic glucose synthesis through the repression of *Pck1* and *G6pc* (21). By targeting Rev-erbα alongside Vdr and Lxr, our therapy likely triggered a more robust autophagic flux, aiding in the clearance of *M. tb* while stabilizing fasting glucose levels.

A key question addressed by this study is whether simultaneous targeting offers advantages over single-target approaches. While individual ligands for these NRs have shown promise, the TB- T2DM comorbid state involves overlapping dysfunctions in lipid signaling, glucose regulation, and cytokine production. Our results suggest a complementary or synergistic effect while Vdr and Rev-erbα bolster the macrophage’s killing capacity, Lxr and Rev-erbα focus on correcting the metabolic environment. This multi-pronged approach potentially allows for the use of lower ligand doses as seen in our dose-reduction strategy thereby minimizing systemic toxicity while maximizing therapeutic efficacy.

We acknowledge that this study focused primarily on the efficacy of the triple combination. A limitation of the current design is the absence of individual or pairwise treatment groups, which would more definitively characterize the degree of synergy between these specific ligands. Future investigations utilizing genetic knockout models or comprehensive monotherapy comparisons are required to fully map the molecular crosstalk between these receptors. Nonetheless, these findings provide a biological proof-of-concept for integrated host-directed therapies in prevalent regions where TB and T2DM co-exist (**Graphical Abstract**).

## Data Availability

The datasets presented in this study can be found in online repositories. The names of the repository/repositories and accession number(s) can be found in the article/**Supplementary Material**.
