# Supplementary figures and images for: Integrated approaches to target nuclear receptors for managing the co-morbidity of tuberculosis and diabetes
Source: Front Immunol. 2026 Jun 10;17:1824354. doi: 10.3389/fimmu.2026.1824354 (PMC13291132; doi:10.3389/fimmu.2026.1824354)

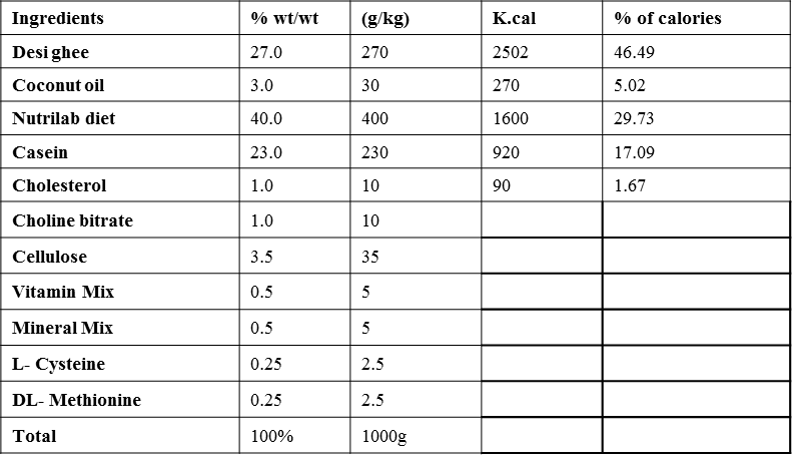

Supplement: Supplementary file 1 [file Image1.tif]
